# Multisystem Inflammatory Syndrome in Children after SARS-CoV-2 Vaccination

**DOI:** 10.3201/eid2805.212418

**Published:** 2022-05

**Authors:** Eisha Jain, Jeffrey R. Donowitz, Elizabeth Aarons, Beth C. Marshall, Michael P. Miller

**Affiliations:** Virginia Commonwealth University School of Medicine, Richmond, Virginia, USA (E. Jain);; Children’s Hospital of Richmond at Virginia Commonwealth University, Richmond (J.R. Donowitz, E. Aarons, B.C. Marshall, M.P. Miller)

**Keywords:** COVID-19, SARS-CoV-2, MIS-C, severe acute respiratory syndrome coronavirus 2, viruses, respiratory infections, zoonoses, COVID-19 vaccine, multisystem inflammatory syndrome in children, vaccines, United States

## Abstract

Multisystem inflammatory syndrome in children (MIS-C) is a hyperinflammatory state that occurs after severe acute respiratory syndrome coronavirus 2 (SARS-CoV-2) infection. We present 2 cases of MIS-C after SARS-CoV-2 vaccination; 1 patient had evidence of recent SARS-CoV-2 infection. Our findings suggest that vaccination modulates the pathogenesis of MIS-C.

Multisystem inflammatory syndrome in children (MIS-C) was first described in 2020 during the severe acute respiratory syndrome coronavirus 2 (SARS-CoV-2) pandemic (*1*). MIS-C is a systemic hyperinflammatory state necessitating hospitalization in patients <21 years of age who experienced >24 hours of fever, recent SARS-CoV-2 exposure or positive testing, involvement of >2 organ systems, and >1 of the following laboratory results: elevated C-reactive protein (CRP), erythrocyte sedimentation rate, fibrinogen, procalcitonin, D-dimer, ferritin, lactate dehydrogenase, interleukin-6, neutrophils, reduced lymphocytes, or reduced albumin (*2*). It is unknown whether vaccination can precipitate or abrogate MIS-C and whether natural infection preceding or at the time of vaccination plays a role (*1*). We describe MIS-C in 2 adolescents recently vaccinated with BNT162b2 (Pfizer-BioNTech, https://www.pfizer.com) and raise the possibility of vaccination modulating MIS-C pathogenesis.

## The Study

Patient 1 was a 15-year-old girl with asthma who received her first dose of BNT162b2 6 days before seeking care. She had low-grade fever and myalgia, which resolved within 2 days of vaccination. Four days later, she experienced 102°F fevers, headaches, nonbilious emesis, myalgias, chest pain, and a rash. Emergency department (ED) examination identified pharyngeal erythema, bilateral conjunctivitis, and a diffuse blanching rash. She had no respiratory or cardiovascular symptoms. At admission, laboratory test results showed leukocytosis with polymorphonuclear cell predominance and elevated CRP, fibrinogen, prothrombin time, brain natriuretic peptide (BNP), and D-dimer (Table). Urinalysis revealed trace protein, large blood, moderate leukocyte esterase, 10–20 leukocytes per high-powered field, and 1+ bacteria. Results of nasopharyngeal SARS-CoV-2 reverse transcription PCR were negative. Further tests included chest radiograph, chest computed tomography angiography, electrocardiogram, and echocardiogram; all results were unremarkable. She was admitted to the pediatric intensive care unit (ICU) and given 2 g/kg intravenous immune globulin (IVIG) for suspected of MIS-C. Symptoms rapidly improved. Leukocyte level decreased to 11.0 K/uL and D-dimer to 2.5 mg/L within 48 hours. The patient remained hemodynamically stable throughout admission and was afebrile with improved symptoms when she was discharged 3 days after admission. SARS-CoV-2 antibody test results at discharge were positive for nucleocapsid but negative for spike. Two days after discharge, the patient returned to the ED for throbbing headaches, nausea, and fatigue. CRP had downtrended since discharge to 2.71 mg/L. Magnetic resonance venography results were normal and she was discharged on antimigraine medication.

**Table:** Data for 2 adolescent patients experiencing multisystem inflammatory syndrome after initial dose of BNT162b2 vaccination against severe acute respiratory syndrome coronavirus 2, United States*

Data	Patient 1	Patient 2
Age, y/sex	15/F	17/F
Underlying conditions	Asthma, seasonal allergies	None
Time since BNT162b2 dose, d	6	7
Initial symptoms	102°F fever, headache, nonbilious emesis, myalgias, chest pain, diffuse blanching rash	103.1°F fever, headache, abdominal tenderness, fatigue, myalgias, and costovertebral angle tenderness
Initial vital signs	Blood pressure 115/56 mm Hg, pulse 105 beats/min, temperature 100.4°F, respiratory rate 20 breaths/min, oxygen saturation 100%, weight 60.8 kg	Blood pressure 104/59 mm Hg, pulse 124 beats/min, temperature 103.1°F, respiratory rate 18 breaths/min, oxygen saturation 99%, weight 58 kg
Laboratory test results (reference range)		
Leukocytes, K/μL (4.2–9.4)	17	21.1
% PMNs (39–74)	91	90
% Lymphocytes (18–50)	3	5
CRP, mg/dL (0–0.60)	15.1	36.7
ESR, mm/H (0–15)	13	83
LDH, U/, sL (130–230)	176	326
Fibrinogen, mg/dL (200–475)	516	>800
Prothrombin time, s (9–11.1)	11.4	11.3
BNP, pg/mL (<125)	169	560
Troponin, ng/mL (<0.05)	<0.05	0.18
D-dimer, mg/L (0–0.65)	2.84	2.58
Creatinine, mg/dL (0.3–1.10)	0.92	1.39
AST U/L (15–37)	16	71
ALT, U/L (12–78)	22	73
Alkaline phosphate, U/L (40–120)	73	258
Additional work-up		
Urinalysis	Trace protein, large blood, moderate leukocyte esterase, 10–20 leukocytes, 1+ bacteria	100 mg/dL of protein, moderate blood, moderate leukocyte esterase, 10–20 leukocytes, 5–10 red blood cells, no bacteria
Urine culture	Not performed	10,000 CFUs *Escherichia coli*
Blood culture	Not performed	Negative
Chest radiograph	No abnormal findings	No abnormal findings
Chest CT	No abnormal findings	Not performed
Electrocardiogram	No abnormal findings	Sinus tachycardia, nonspecific T-wave abnormalities
Echocardiogram	No abnormal findings	No abnormal findings
Abdomen/pelvis CT	Not performed	Diffuse left renal enlargement, possible polycystic ovaries
COVID-19 labs		
Nasopharyngeal RT-PCR	Negative	Negative
Spike antibody	Negative	Positive
Nucleocapsid antibody	Positive	Not performed
PICU admission	Yes	No
Treatment	2 g/kg IVIG	2 g/kg IVIG for 1 d, 30 mg IV methylprednisolone 2×/d for 3 d to continue at home orally for 2 d then 2–3 wk steroid taper, 325 mg aspirin reduced to 81 mg on day 3, cefdinir 7 d course
Length of hospital stay	1 d	3 d

*BNT162b2, Pfizer-BioNTech (https://www.pfizer.com). ALT, alanine transaminase; AST, aspartate transaminase; BNP, brain natriuretic peptide; COVID-19, coronavirus disease; CRP, C-reactive protein; CT, computed tomography; ESR, erythrocyte sedimentation rate; IVIG, intravenous immune globulin; LDH, lactate dehydrogenase; PICU, pediatric intensive care unit; PMN, polymorphonuclear cells; RT-PCR, reverse transcription PCR.

Patient 2 was a previously healthy female 17-year-old who received her first dose of BNT162b2 vaccination 7 days before seeking care. Three days after vaccination, she experienced fevers, headaches, abdominal pain, fatigue, and myalgias. Her primary care provider noted leukocytosis to 20 K/uL and referred her to the ED. She had a 103.1°F fever, diffuse abdominal tenderness, and costovertebral angle tenderness. She had no respiratory symptoms. At admission, laboratory test results showed leukocytosis with polymorphonuclear cell predominance and elevated CRP, erythrocyte sedimentation rate, lactate dehydrogenase, BNP, troponin, D-dimer, creatinine, aspartate aminotransferase, and alkaline phosphate (Table). Urinalysis revealed 100 mg/dL protein, moderate blood, moderate leukocyte esterase, 10–20 leukocytes per high-powered field, 5–10 red blood cells per high powered field, and no bacteria. Urine culture was positive for 10,000 CFU/mL of *Escherichia coli*. Blood culture results were negative. Electrocardiogram showed sinus tachycardia and nonspecific T-wave abnormalities. Abdomen and pelvis computed tomography showed diffuse left renal enlargement without hypoattenuation or hyperattenuation and possible polycystic ovaries. Results of chest radiograph and echocardiogram were normal. Nasopharyngeal SARS-CoV-2 RT-PCR was negative. Results of SARS-CoV-2 spike antibody testing were positive; nucleocapsid antibody testing was not performed. She started 3 days of intravenous methylprednisolone (30 mg 2×/d) and 1 day IVIG (2 g/kg) for MIS-C. Troponin decreased to <0.05 within 24 hours and CRP to 16.2 within 48 hours. BNP rose to 2,024 on hospital day 2. Repeat echocardiogram showed mild right coronary artery ectasia, and she was started on 325 mg of aspirin daily. On hospital day 3, repeat echocardiogram results were normal, and she was afebrile. Aspirin was decreased to 81 mg daily. She was discharged on hospital day 4 with no fevers for 60 hours and downtrending inflammatory markers including CRP to 8.49 mg/dL. She was also treated for a possible UTI.

## Conclusions

This report describes 2 cases of MIS-C within 1 week of receiving the first dose of BNT162b2. There is no specific test for MIS-C; although both patients met diagnostic criteria, alternative diagnoses were possible. Patient 2 had costovertebral angle tenderness, unilateral renal enlargement, and 10,000 CFU/mL growth of a uropathogen on culture. Given the low level of bacterial growth, lack of enhancement on her CT, and constellation of lab and imaging abnormalities not commonly seen with urinary tract infections, MIS-C remains her most likely diagnosis.

Patient 1 had a positive antinucleocapsid antibody suggesting community-acquired COVID-19 infection before MIS-C developed (P.D. Burbelo et al., unpub. data, https://doi.org/10.1101/2020.04.20.20071423). Salzman et al. describe 3 similar cases in which MIS or an MIS-like illness developed after COVID-19 vaccination, particularly in the setting of community-acquired COVID-19 (*3*). The chronology of events in these cases raises the possibility that vaccination may be involved in the pathogenesis of MIS-C when preceded by community-acquired SARS-CoV-2.

The pathogenesis of MIS-C is thought to involve immune dysregulation and hyperinflammation (*4*). Studies have identified high levels of receptor-binding protein (RBD) antibodies in children with severe MIS-C (*5**,**6*). Both natural SARS-CoV-2 infection and BNT162b2 vaccination have been shown to elicit RBD antibodies (*7*). It may be possible that the immune responses to these 2 forms of exposure to SARS-CoV-2 interact to shape the manifestations of mild MIS-C in the postinfectious period of COVID-19. Although both of these cases were mild, we have insufficient data on the pathogenesis of MIS-C to understand how vaccination may shape symptomatology.

A recent report by Zambrano et al. documented that 61/97 (62.9%) MIS-C cases in unvaccinated patients required ICU admission (*8*). That report had a small number of vaccinated cases; 1 in 5 of those vaccinated needed ICU care (*8*). An analysis of postvaccination MIS-C in 21 patients showed that 3 (14%) required invasive mechanical ventilation, 8 (38%) required vasopressors, and 12 (57%) required ICU care (A.R. Yousaf et al., unpub. data, https://doi.org/10.1101/2022.01.03.22268681). In contrast to Zambrano et al.’s vaccinated cases and our reported cases, the Yousaf et al. report suggests a similar number of ICU admissions in vaccinated and unvaccinated persons. 

Studies have shown that COVID-19 vaccination is associated with reduced incidence of MIS-C, especially if 2 doses are given. A study of MIS-C cases in France during September–October 2021 found a significantly lower risk of MIS-C among vaccinated adolescents than those who were unvaccinated (*9*). Zambrano et al. found a 91% protective effect of complete (2 doses) BNT162b2 vaccination against MIS-C (*8*). Phase 2 and phase 3 clinical trials of BNT162b2 revealed 0 cases of MIS-C after vaccination (*10*). Despite the reports of postvaccination MIS-C, vaccination clearly lowers the overall MIS-C burden, probably by preventing infection. These studies also suggest low likelihood of vaccination triggering development of MIS-C. 

If vaccination can play a role in MIS-C pathogenesis, it is likely an extremely rare event and may involve an underlying genetic predisposition or be contingent on extraneous factors like recent SARS-CoV-2 community exposure. Our findings in 2 cases of MIS-C within 1 week of a dose of BNT162b2 raise the possibility that vaccination may alter the symptom profile of MIS-C. 
